# Unexpected Giant Right Coronary Artery Aneurysm Diagnosed by Computed Tomography Angiography in the Emergency Department

**DOI:** 10.1002/jcu.70083

**Published:** 2025-09-21

**Authors:** Stefano Giusto Picchi, Giulia Lassandro, Rosita Comune, Filomena Pezzullo, Stefania Tamburrini, Giulio Cocco, Nino Cocco, Domenico Tafuri, Antonio Corvino

**Affiliations:** ^1^ Department of Radiology Ospedale del Mare‐ASL NA1 Centro Naples Italy; ^2^ Department of Precision Medicine, Section of Radiology and Radiotherapy University of Campania “Luigi Vanvitelli” Naples Italy; ^3^ Department of Neuroscience, Imaging and Clinical Sciences University “G. d'Annunzio” Chieti Italy; ^4^ Department of Cardiovascular Sciences Campus Bio‐Medico University of Rome Rome Italy; ^5^ Medical, Movement and Wellbeing Sciences Department University of Naples “Parthenope” Naples Italy

**Keywords:** 3D reconstructions CCTA, computed tomography angiography (CTA), coronary CTA (CCTA), emergency setting, giant right coronary artery aneurysm (GCAA)

## Abstract

Giant coronary artery aneurysms (GCAA) are usually defined as diameter > 8 mm or > 400% of the adjacent normal segment; they are very rare (reported prevalence ≈0.02%). Though coronary angiography is the diagnostic gold standard, computed tomography angiography (CTA) offers a non‐invasive, highly sensitive, and specific alternative. CTA enables detailed visualization of aneurysm morphology and detection of complications. We present the case of a 72‐year‐old man admitted to the Emergency Department with chest pain, where CTA played a crucial role in diagnosing a GCAA and assessing its potential life‐threatening complications, highlighting its value in emergency cardiovascular imaging.

## Graphical abstract

3D Volume Rendering coronary CTA reconstruction showing a giant thrombosed aneurysm of the proximal right coronary artery

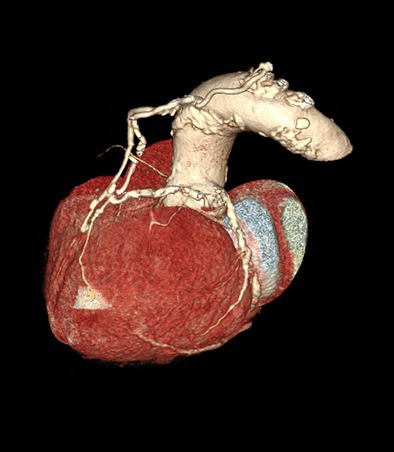

**Abbreviations**
CAA: coronary artery aneurysm
CCTA: coronary CTA
CTA: computed tomography angiography
GCAA: giant coronary artery aneurysm
LAD: left anterior descending
RCA: right coronary artery

## 1 Introduction

An aneurysm is defined as a focal dilatation of a vessel ≥ 1.5 times the adjacent normal segment, due to intrinsic weakness of the vessel wall (Shaw et al. 2023).

Coronary artery aneurysm (CAA) is an uncommon entity, found in 0.3%–5% of patients undergoing coronary angiography (Crawley et al. 2014). CAA can be termed “giant” (GCAA) if the dilated segment has a diameter > 8 mm or 400% of the diameter of adjacent segments (Khouzam and Khouzam 2021; Kato et al. 1996), and this entity has an incidence of 0.02% in the general population (Pham et al. 2020; Li et al. 2005).

Right coronary artery (RCA) aneurysm is the most common aneurysmal finding (40%–87%), followed by dilatation of the left anterior descending (LAD) coronary artery and left circumflex artery (Pham et al. 2020; Swaye et al. 1983).

The etiology of CAAs is not entirely clear, but the most common causes include congenital heart disease, atherosclerosis, myocardial infarctions, vasculitis, and systemic inflammatory conditions (e.g., Kawasaki disease, Takayasu arteritis, systemic lupus erythematosus), connective tissue disorders (e.g., Marfan syndrome, Ehlers‐Danlos syndrome), drug‐related causes (cocaine, amphetamines, protease inhibitors), iatrogenic and traumatic causes (percutaneous coronary intervention, angiography), infections, and idiopathic causes (Crawley et al. 2014; Tinson et al. 2023).

Patients with CAAs may be completely asymptomatic, and CAAs are often incidental findings during coronarography or computed tomography angiography (CTA); when symptomatic, they manifest with acute coronary syndrome, angina pectoris, or dyspnea, or they may be revealed in the presence of related complications, such as fistula, cardiac compression, tamponade, congestive heart failure, rupture, or external compression on surrounding structures (Pham et al. 2020; Tinson et al. 2023). GCAAs are most often symptomatic and sometimes may also mimic a mediastinal mass or cardiac tumor (DadkhahTirani et al. 2016).

Coronary angiography is the gold standard for the diagnosis and evaluation of GCAs, but CCTA is a non‐invasive diagnostic method that allows imaging of the anatomy of coronary arteries, detection of coronary aneurysms by evaluating their shape and structure, and excluding potential complications, proving to be highly sensitive and specific for coronary aneurysms evaluation (Díaz‐Zamudio et al. 2009).

Here we report a rare case of a 72‐year‐old man who presented in the Emergency Department with severe chest pain. The purpose of our article is to demonstrate how CTA represents a highly sensitive and specific radiologic imaging test for the diagnosis of coronary aneurysms in the Emergency Department and is also useful in identifying potential even lethal complications.

## 2 Case Report

A 72‐year‐old man presented to the Emergency Department of our center (Ospedale del Mare, Naples, Italy) with severe chest pain and dyspnea for 1 h. His medical history was positive for diabetes mellitus, hypertension, hypokinetic heart disease undergoing myocardial revascularization surgery by coronary artery bypass 20 years earlier, permanent atrial fibrillation awaiting cardiac pacemaker implantation, moderate arterial stenosis, and mild mitral stenosis.

On arrival, his vital signs were as follows: blood pressure 140/80 mmHg, heart rate 108 beats/min, respiratory rate 22 breaths/min, temperature 36.5°C, and oxygen saturation 88% on room air; therefore, oxygen therapy was set. Physical examination revealed decreased vesicular breath sounds bilaterally, pure cardiac tones, a 2/6 systolic murmur, an irregularly irregular rhythm consistent with atrial fibrillation, and dependent edema of the lower extremities.

Routine blood tests revealed a slight increase in white blood cells (12.6 10^3^/μL; range: 4.05–11.0 10^3^/μL), predominantly neutrophils, a slight decrease in hemoglobin (11.2 g/dL; range: 13.4–16.7 g/dL) and hematocrit (35.2%; range: 39.2%–48.6%), increased blood glucose (153 mg/dL; range: 74–110 mg/dL), increased d‐dimers (4793 μg/L FEU; range: 0–500 μg/L FEU), and increased cardiac necrosis markers such as troponin T (0.559 ng/mL; range: 0.003–0.014 ng/mL) and Creatine Kinase‐MB (20.6 ng/mL; range: 0–4.87 ng/mL).

**FIGURE 1:**
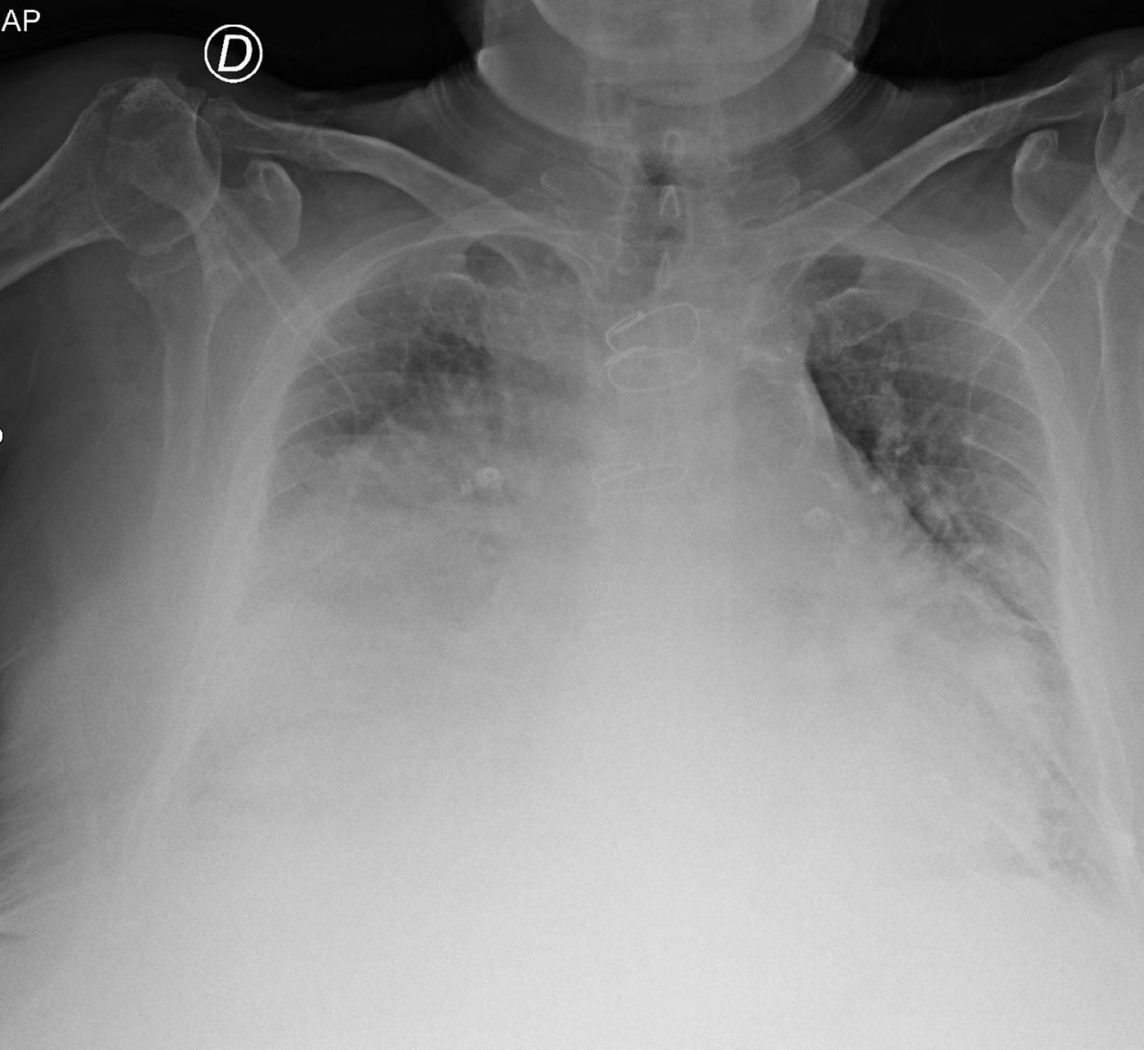
Bedside chest X‐ray showing bilateral pleural effusion with congested appearance of the pulmonary hila and a broad opacity enlarging the right middle–basal cardiac silhouette, suspicious for mass or cardiomegaly.

The patient ECG confirmed atrial fibrillation with signs of anterolateral myocardial ischemia. A bedside chest X‐ray was performed, which showed bilateral pleural effusion with congested appearance of the pulmonary hila and a broad opacity enlarging the right middle–basal cardiac silhouette, suggestive of cardiomegaly or mass (Figure 1), raising the suspicion of heart failure with pulmonary edema or a mediastinal mass‐like lesion.

Subsequently, the patient underwent cardiac ultrasonography, which showed a non‐dilated left ventricle with an ejection fraction of 40%, basal inferior myocardial hypokinesis, and a basal inferior interventricular septal defect. In addition, a compressive determinant formation on the right atrium was evident, necessitating further investigation by urgent chest CTA.

CTA scan was performed using a 128‐slice multidetector CT scanner (Siemens Healthineers, Forchheim, Germany). Non‐contrast, arterial, venous, and delayed phase were included in the emergency protocol. Acquisition parameters were as follows: rotation time 0.33 s, collimation 128 × 0.6 mm, slice thickness 1.0 mm, reconstruction interval 0.8 mm, tube voltage 120 kVp, and tube current with automatic exposure modulation. The intravenous contrast medium was Iomeprol 400 mg I/mL, 1.0–1.5 mL/kg, which was injected at 4.0 mL/s, followed by a 50 mL saline flush (bolus tracking in the ascending aorta at a threshold of 120 HU). A dedicated workstation was used for post‐processing, obtaining multiplanar reconstructions, volume rendering 3D technique reconstructions, and maximum intensity projections.

**FIGURE 2:**
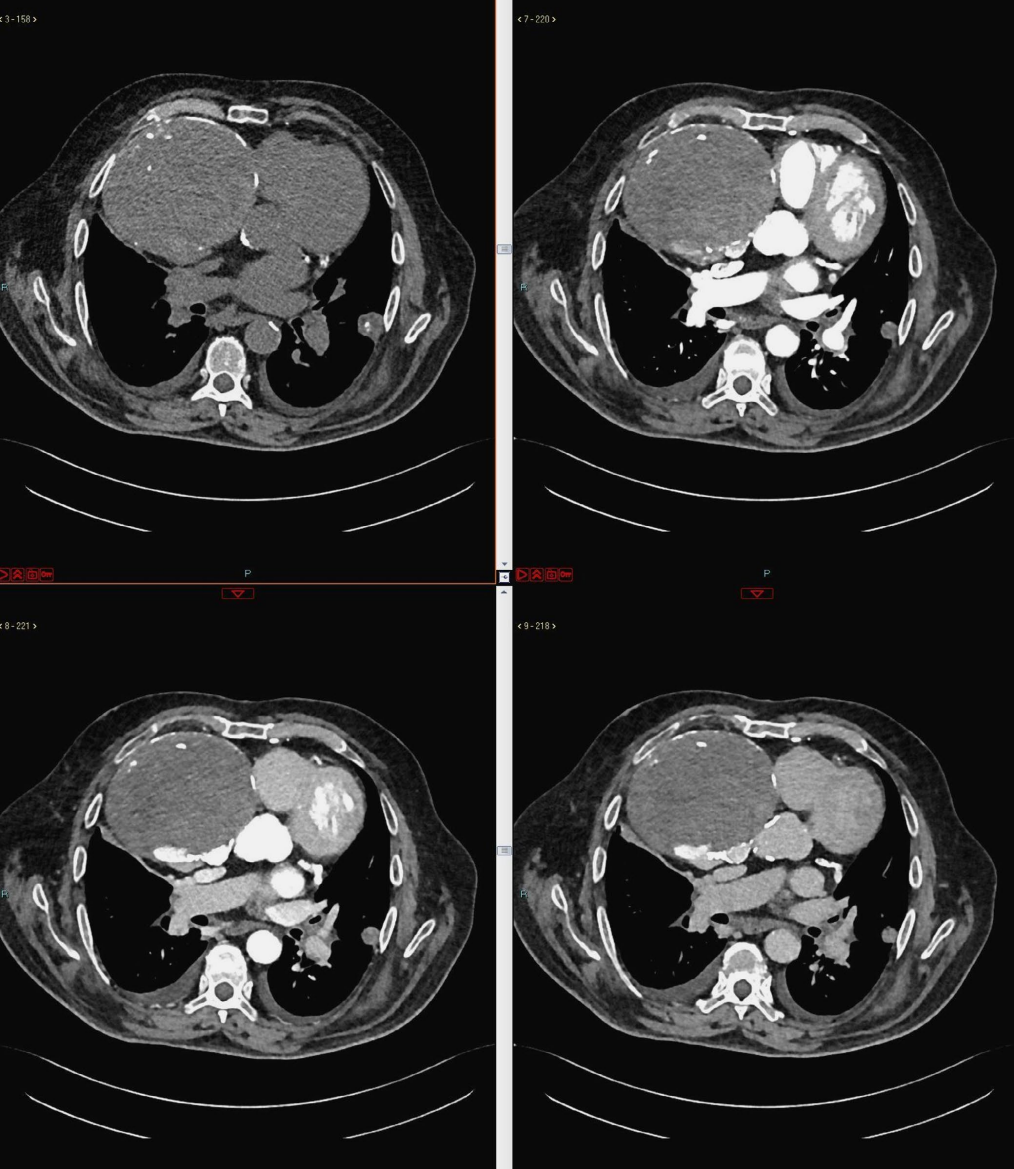
Computed tomography angiography (CTA), axial planes, non‐contrast (upper left), arterial (upper right), venous (lower left), and delayed (lower right) contrast‐enhanced phases. CTA scan shows a large formation (16 × 14 × 13 cm) with parietal and intralesional calcifications, predominantly hypodense in the non‐contrast scan and contrast‐enhanced in the delayed phases, causing right atrium compression.

**FIGURE 3:**
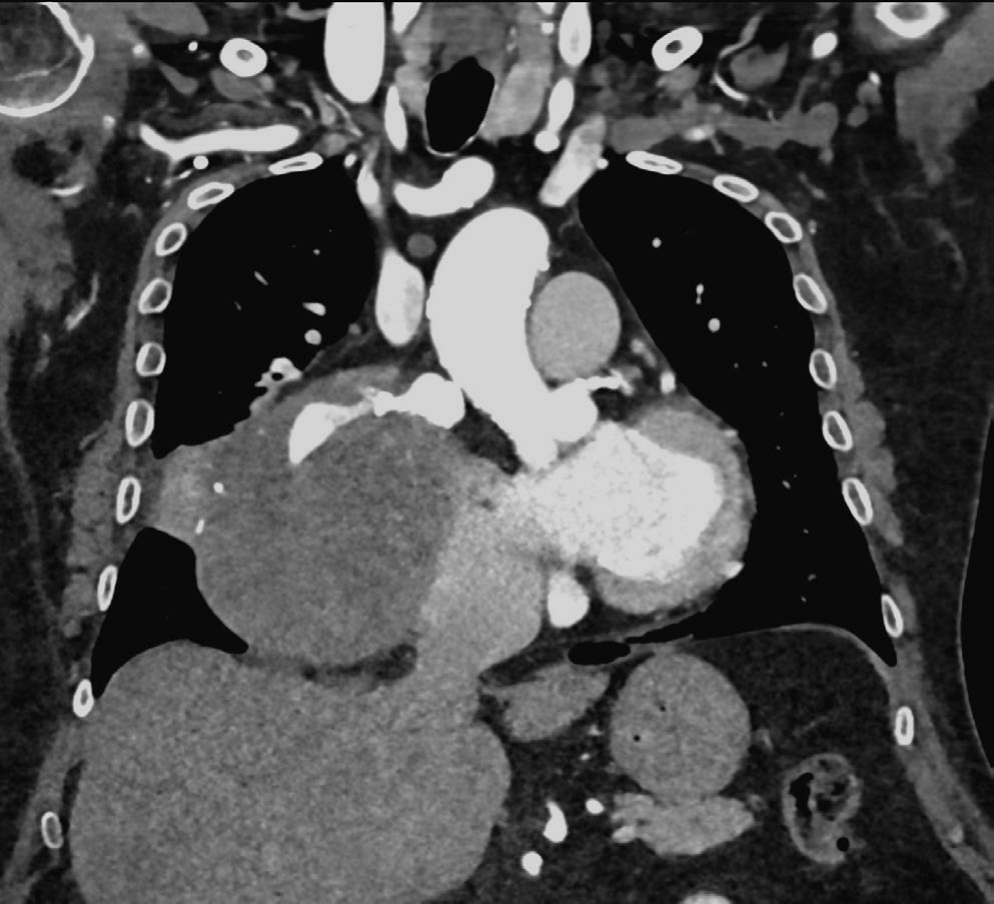
CTA, coronal plane, arterial phase. The communication with the right coronary artery (RCA) proximal tract can be noticed.

**FIGURE 4:**
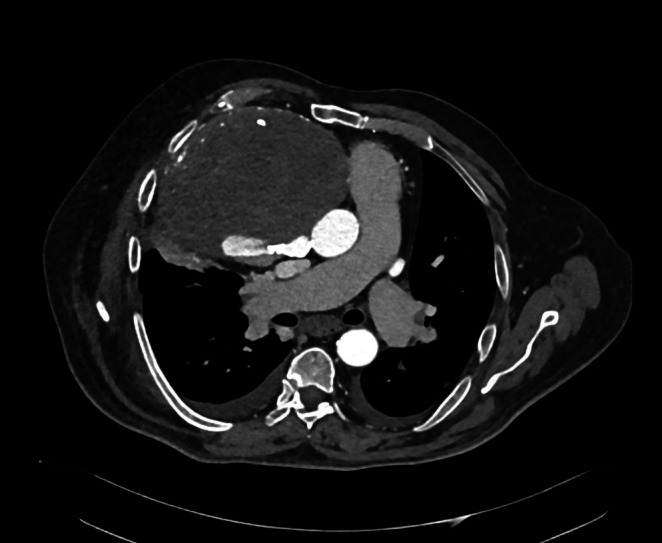
CTA, axial plane, arterial phase. The communication with RCA proximal tract can be noticed.

CTA scan showed a large formation characterized by parietal and intralesional calcifications measuring approximately 16 × 14 × 13 cm, with maximum diameters obtained in the three orthogonal planes by using axial and multiplanar reconstruction images; it was predominantly hypodense but with contrast enhancement in late acquisition phases, causing right atrial compression. Thus, the diagnosis of thrombosed GCAA of the proximal RCA was posed (Figures 2, 3, 4). The aneurysm was considered to result from a slowly progressive process that had developed over a long period before the emergency presentation, owing to its unusual size and extensive calcifications, even though no earlier imaging examinations were available to confirm this. As additional findings, CTA also revealed bilateral pleural effusion with basal parenchymal atelectasis, bilateral ground‐glass opacities with interstitial thickening, and cardiomegaly, configuring a picture of acute interstitial–alveolar pulmonary edema sustained by heart failure.

The patient was admitted to the NICU and treated pharmacologically with anticoagulant and antiplatelet therapy, which was chosen because of the existing permanent atrial fibrillation, prior ischemic heart disease, and the additional thromboembolic risk related to the GCAA. Additionally, he received diuretics such as loop diuretic and spironolactone, a β‐blocker selective for the β‐1 receptor, oral antidiabetic therapy, hypolipidemic agents, antibiotics, and sodium chloride (NaCl) 0.9%.

**FIGURE 5:**
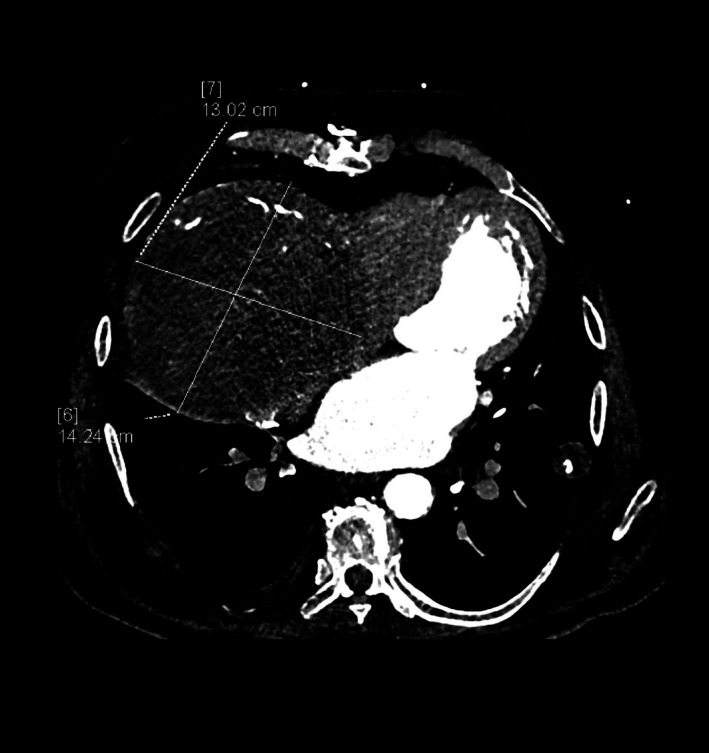
Coronary CTA, confirming giant coronary artery aneurysm (GCAA) of RCA.

**FIGURE 6:**
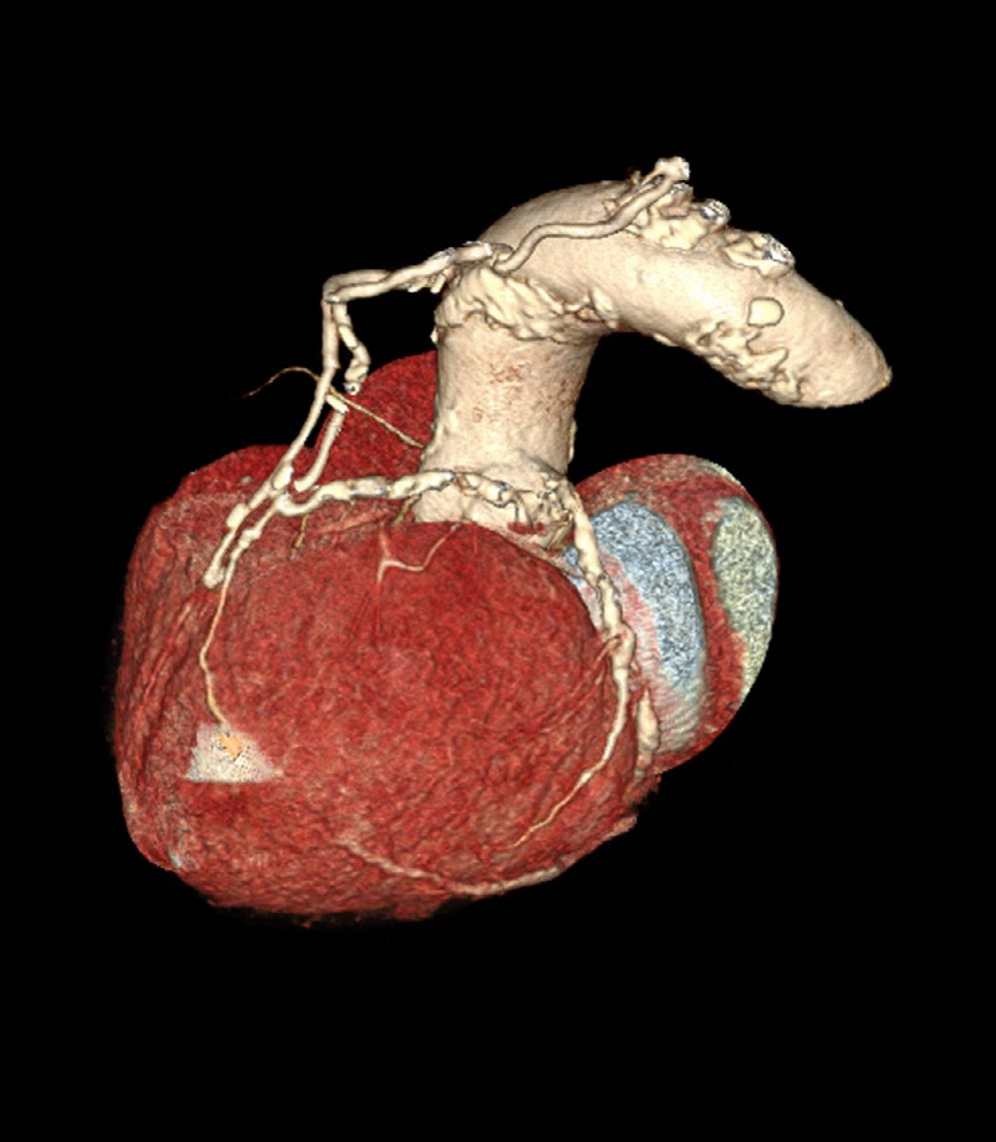
Coronary CTA, 3D reconstructions by dedicated software, confirming GCAA of RCA.

During hospitalization and after stabilization, a dedicated coronary CTA (CCTA) was performed using the same 128‐slice CT scanner, with retrospective ECG‐gated acquisition and a thinner slice thickness (0.75 mm) for detailed coronary artery evaluation. GCAA of the proximal tract of the RCA was confirmed, with a mostly thrombosed lumen and signs of late perfusion in the sac with a predominantly posterior and superior distribution (Figures 5 and 6).

Although invasive coronary angiography is traditionally considered the gold standard for coronary aneurysm evaluation, in this case, it was not performed after stabilization. This decision was made because CCTA had already provided a thorough evaluation, and the patient was at significant procedural risk. Therefore, the patient was proposed for cardiac surgery, which he refused despite being informed of the need for the procedure and of the risks associated with refusal. No indication was placed for percutaneous revascularization because of an unfavorable risk/benefit ratio. After complete stabilization of the clinical setting, the patient was discharged with appropriate medical therapy and scheduled cardiologic follow‐up at 1 month.

## 3 Discussion

CAA is an uncommon and incidentally found entity found in 0.3%–5% of patients undergoing coronary angiography (Crawley et al. 2014). CAA is considered giant if the dilated segment is either > 8 mm in diameter or 400% of the diameter of the adjacent segments that is even rarer, with an incidence of 0.02% in the general population (Pham et al. 2020; Li et al. 2005). RCA disease is the most prevalent CAA, involved in 40%–70% of cases, followed by the LAD (35%) and the left circumflex artery with 25% of cases (El Khoury et al. 2021).

The etiology of CAAs is not entirely clear, and there are a lot of different causes, including atherosclerosis as the most common in adults, accounting for up to 50% of cases, and Kawasaki disease and Takayasu arteritis in the pediatric population (Morita et al. 2012).

The pathological mechanism of CAAs remains controversial: an intrinsic defect in the vessel wall may predispose atherosclerotic vessels to aneurysm formation: the vessel wall media is weakened, with a focal decrease in elasticity and reduced tolerance to endoluminal pressure, resulting in vessel dilatation (Crawley et al. 2014; Tinson et al. 2023).

Clinically, CAAs are usually asymptomatic and often incidental findings, whereas when symptomatic, clinical presentations include angina, myocardial infarction, congestive heart failure, or sometimes sudden death; GCAAs are more often symptomatic (Johnson and Fishman 2010; Cohen and O'Gara 2008). Often, when CAAs are clinically symptomatic and more frequently in the case of GCAAs, complications can be associated, which are: local thrombosis, distal embolization, cardiac compression, tamponade, rupture, and external compression on surrounding structures (Pham et al. 2020; Tinson et al. 2023; DadkhahTirani et al. 2016).

A variety of imaging techniques have been used to view CAAs: non‐invasive exams such as echocardiography, chest radiography, CT (CTA and/or CCTA), and magnetic resonance may detect CAAs, but coronary angiography is the gold standard for the diagnosis (Corvino et al. 2020, 2024; Eshtehardi et al. 2008; Chrissoheris et al. 2008).

Chest radiography is non‐specific for detecting CAAs; in fact, the radiograph may show radiopaque masses in the paracardiac site of non‐univocal interpretation, without being able to define their exact attribution (Pham et al. 2020; Li et al. 2005).

Coronary angiography is the “gold standard” technique for the diagnosis and evaluation of CAA, as it provides information about the anatomy of the aneurysm (size, shape, number, and location) and associated coronary artery disease, such as atherosclerosis. In addition, it allows comprehensive evaluation of the coronary arteries (stenosis, collateral arteries, anatomical changes, etc.) (Pham et al. 2020). The main limitation of coronary angiography is the underestimation of aneurysm size in the case of intraluminal thrombi and the inability to detect an abnormality of the wall artery. In fact, delay in anterograde contrast filling and contrast stagnation in the dilated coronary segment may hinder optimal imaging of coronary angiography (Pham et al. 2020).

CCTA is highly sensitive and specific for CAAs, with a sensitivity of 100% to detect aneurysms and a sensitivity of 87.5% and specificity of 92.5% to detect significant coronary stenosis (Kanamaru et al. 2005), partly due to the use of multiplanar reconstruction algorithms in the three planes of space, 3D volumetric rendering, and maximum intensity projection. CCTA provides important information about the size, shape, location, and number of aneurysms. It can also reveal potential related complications, such as fistula, cardiac compression, tamponade, congestive heart failure, rupture, or external compression on surrounding structures (Pham et al. 2020; Tinson et al. 2023).

In the literature, most GCAAs measure between 2 and 10 cm, whereas in our patient the aneurysm reached 16 × 15 × 14 cm, placing it among the largest cases described (Ali et al. 2023; Kurnauth et al. 2020). Similarly to other reports, clinical presentation was related to mass effect and heart failure rather than acute coronary syndrome, and the complications detected were extensive thrombosis and right atrial compression (Ali et al. 2023; Kurnauth et al. 2020; Corvino et al. 2019).

The differential diagnosis of a mediastinal mass mainly includes solid tumors (thymoma, lymphoma, or germ cell tumors), cysts, and vascular lesions (Ahuja et al. 2023). In our case, CTA ruled out neoplastic and cystic masses, easily confirming the vascular nature of the lesion. CTA not only identifies and characterizes coronary aneurysms, but also provides high‐resolution anatomical and functional information that is crucial in multiple acute scenarios, particularly of vascular origin. In these contexts, the rapid and accurate evaluation of vascular and extravascular structures provided by CTA can be decisive and even life‐saving (Parillo et al. 2024, 2023). For example, in cardiac perforation, CTA can detect pericardial effusion, active extravasation as well as the relationships with adjacent cardiac chambers, thus guiding emergency management (Parillo et al. 2024). Similarly, in coronary artery dissection, CTA enables a non‐invasive assessment of the dissected artery, the presence of intramural hematoma, and downstream ischemia, thus integrating invasive angiography and supporting therapeutic decision‐making (Parillo et al. 2023).

Management options for CAA include: medical therapy with anticoagulation/antiplatelet agents and risk‐factor management (usually reserved for asymptomatic patients or for those ineligible for invasive procedures); percutaneous treatment with covered stent implantation (feasible only when the aneurysm is small, saccular, with a short and well‐defined neck and limited thrombus); coil or plug embolization in selected cases (typically when the aneurysm does not involve a major coronary vessel or when collateral circulation ensures adequate myocardial perfusion); and surgery, most often consisting of aneurysm resection or exclusion combined with coronary bypass grafting (which remains the preferred strategy in large, thrombosed, symptomatic, or complicated cases, as in our patient) (Kawsara et al. 2018; Zhu et al. 2021).

In our case, a percutaneous approach was not pursued after stabilization because of several unfavorable features: aneurysm size, thrombosed lumen, absence of a discrete neck suitable for stent placement, proximal RCA involvement with mass effect, and the patient's comorbidities. For these reasons, surgical exclusion with bypass grafting was considered the most appropriate definitive treatment, but the patient refused surgery and was therefore managed conservatively with optimized medical therapy.

The prognosis for GCAA is usually negative when not treated, resulting in an increased risk of progressive thrombosis, distal embolization, rupture, and sudden death, especially in lesions of this size (Kawsara et al. 2018). The significance of conservative management is essentially palliative, focusing on symptoms and prevention of thromboembolic events. In these cases, it is essential to perform a close clinical and imaging follow‐up, including checks at 1, 3, and 6 months, and subsequently every 6–12 months, to promptly identify any progression or new complications (Kawsara et al. 2018; Zhu et al. 2021).

## 4 Conclusion

CAAs are an uncommon entity, and their giant form is very rare. Patients with GCAA are at high risk of complications and should benefit from medical therapy and aneurysm exclusion, either by percutaneous or surgical techniques. CCTA is a valuable non‐invasive tool that complements invasive angiography for diagnosis, characterization, and preoperative planning of CAAs. With the increasing use of CCTA, radiologists need to be aware of predisposing conditions and diagnostic criteria for coronary aneurysm to rapidly guide management and avert complications.

## Ethics Statement

The research was conducted according to the guidelines of the Declaration of Helsinki. Authors declare that all the procedures being performed were part of the routine care, all the collected data were anonymized, and no information is linked or linkable to a specific person.

## Consent

Written informed consent was obtained from the patient and is available on request from the first author [S.G.P.].

## Conflicts of Interest

The authors declare no conflicts of interest.

## Data Availability

The data that support the findings of this report are available on request from the first author [S.G.P.]. The data are not publicly available due to restrictions that could compromise the privacy of patient.
